# Pressure-induced changes on the morphology and gene expression in mammalian cells

**DOI:** 10.1242/bio.058544

**Published:** 2021-07-14

**Authors:** Kazuko Okamoto, Tomonobu M. Watanabe, Masanobu Horie, Masayoshi Nishiyama, Yoshie Harada, Hideaki Fujita

**Affiliations:** 1 RIKEN Center for Biosystems Dynamics Research, Laboratory for Comprehensive Bioimaging, Kobe, Hyogo 650-0047, Japan; 2 Department of Stem Cell Biology, Research Institute for Radiation Biology and Medicine, Hiroshima University, Hiroshima, Hiroshima 734-8553, Japan; 3 Radioisotope Research Center, Division of biochemical engineering, Kyoto University, Kyoto, Kyoto 606-8501, Japan; 4 Department of Physics, Faculty of Science and Engineering, Kindai University, Higashi-Osaka, Osaka 577-8502, Japan; 5 Institute for Protein Research, Laboratory of Nanobiology, Osaka University, Suita, Osaka 565-0871, Japan

**Keywords:** Mechanobiology, Pluripotency, Mouse embryonic fibroblast, Embryonic stem cells

## Abstract

We evaluated the effect of high hydrostatic pressure on mouse embryonic fibroblasts (MEFs) and mouse embryonic stem (ES) cells. Hydrostatic pressures of 15, 30, 60, and 90 MPa were applied for 10 min, and changes in gene expression were evaluated. Among genes related to mechanical stimuli, death-associated protein 3 was upregulated in MEF subjected to 90 MPa pressure; however, other genes known to be upregulated by mechanical stimuli did not change significantly. Genes related to cell differentiation did not show a large change in expression. On the other hand, genes related to pluripotency, such as *Oct4* and *Sox2*, showed a twofold increase in expression upon application of 60 MPa hydrostatic pressure for 10 min. Although these changes did not persist after overnight culture, cells that were pressurized to 15 MPa showed an increase in pluripotency genes after overnight culture. When mouse ES cells were pressurized, they also showed an increase in the expression of pluripotency genes. These results show that hydrostatic pressure activates pluripotency genes in mammalian cells.

This article has an associated First Person interview with the first author of the paper.

## Abstract (precis)

**Summary:** Application of high hydrostatic pressure on somatic cells induce changes in gene expression including upregulation in pluripotency genes.

## INTRODUCTION

Mechanosensing is a biogenic function across biological species from bacteria to higher organisms. Various cells and tissues sense and respond to mechanical stimuli received from the surrounding environment (Wang et al., 2008; Kobayashi and Sokabe, 2010; Mammoto et al., 2013; Ivanovska et al., 2015). It has been reported that stimuli change various cellular reactions, such as the signaling process at the membrane and protein expression levels. It eventually has a significant influence on cell development and differentiation (Engler et al., 2006; Chowdhury et al., 2010). Effects of mechanical stimuli on cells are usually studied in cells and tissues that were attached to the substrate in advance, and then mechanical stimuli were applied to the research targets. Representative examples of applied stimuli include flow shear stress and cyclic stretching of soft substrates. However, these methods have certain limitations, in that attachment of the biological samples to the substrate itself could become a mechanical stimulus. For example, it is known that the stiffness of the substrate can change the differentiation of stem cells (Engler et al., 2006). Therefore, the evaluation of the effects of subsequent stimuli seems to be difficult.

On the other hand, we have focused on hydrostatic pressure as a new method to apply mechanical stimuli to biological samples. High-pressure techniques have been used to investigate the effect of hydrostatic pressure on mammalian cells (Salmon et al., 1976; Koyama et al., 2001; Frey et al., 2006; Wagner et al., 2008). The application of pressure causes significant changes in cell morphology and activity. Recently, it has been reported that the application of hydrostatic pressures improves aging progression through the activation of DAF-16/FOXO in *Caenorhabditis elegans* (Watanabe et al., 2020). In mammalian cells, it has been reported that hydrostatic pressure induces changes in gene expression, altering gene regulatory networks (Takahashi et al., 1998; Karjalainen et al., 2003; Karamesinis et al., 2017; Burton et al., 2020). It has also been shown that hydrostatic pressure application to oocytes or embryos improve developmental competence and cryotolerance (Du et al., 2008; Bock et al., 2010), implying that hydrostatic pressure may influence stem cell gene regulation. However, little is known about the pressure stimuli to cellular behaviors because of the lack of study using different pressure methods in this research field.

In order to investigate the effect of pressure stimuli on cells, we observed the morphology and gene expression of mouse embryonic fibroblasts (MEFs) and mouse embryonic stem (ES) cells under high hydrostatic pressure. The morphological change was observed using a recently developed high-pressure microscope (Nishiyama, 2017). To further evaluate the effect of hydrostatic pressure, we conducted gene expression analysis. We found that genes expression changes were not only prone to mechanical stimuli, but some that were related to pluripotency were also upregulated. Upregulation of pluripotency genes was also observed in pressurized ES cells. These results show the possibility of hydrostatic pressure treatment in mammalian cells for gene expression control.

## RESULTS AND DISCUSSION

### Application of hydrostatic pressure on fibroblasts

**Fig. 1.**
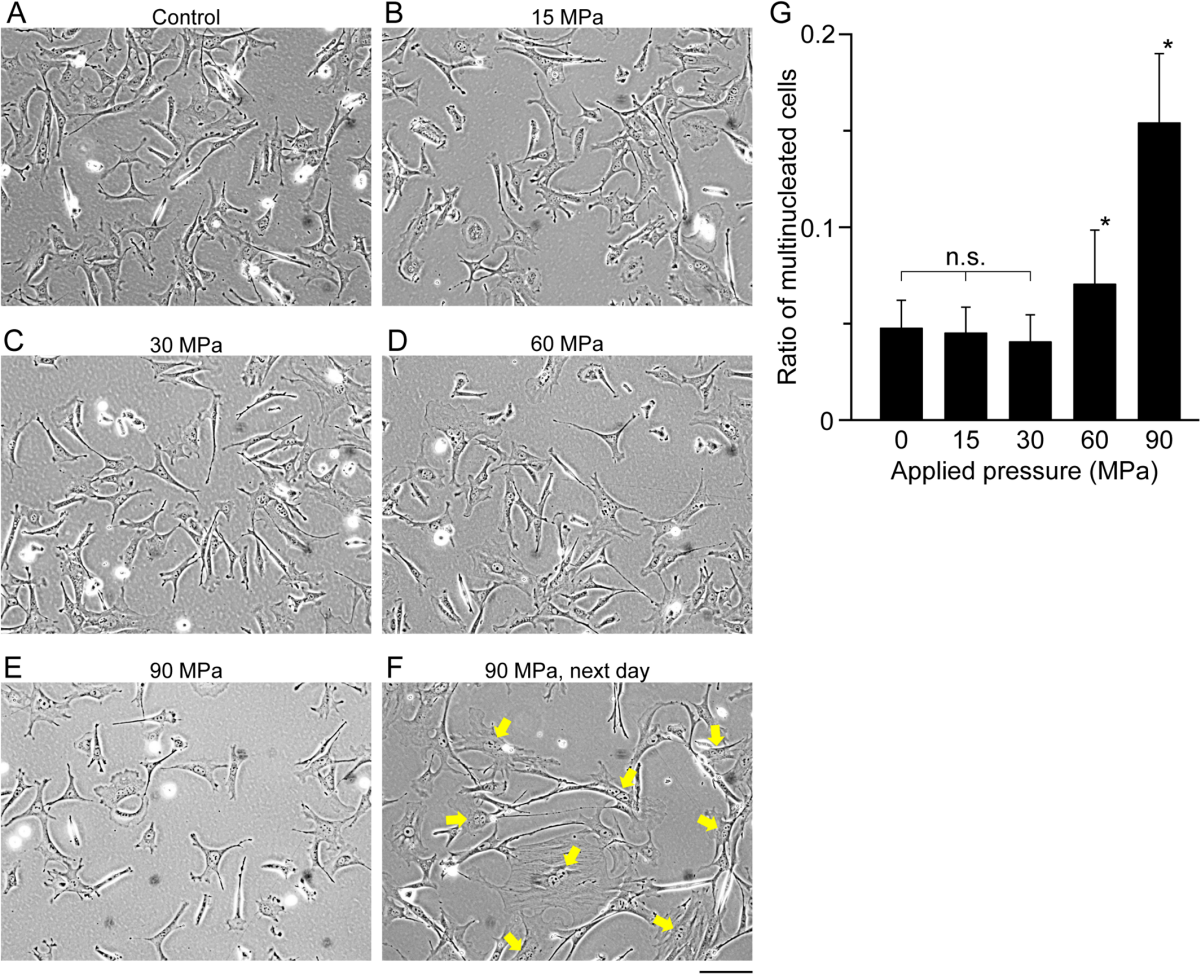
**Phase contrast images of MEF cells subjected to hydrostatic pressure and plated on culture dish for 6 h.** Control MEF cells (A), 15 MPa pressurized cells (B), 30 MPa pressurized cells (C), 60 MPa pressurized cells (D), and 90 MPa pressurized cells (E) are shown. Pressure was applied for 10 min. (F) Cells pressurized to 90 MPa and cultured overnight. Cells having two nuclei are indicated by arrows. Scale bar: 100 μm. (G) Ratio of multinucleated cells cultured overnight after pressure application. Asterisks show statistical significance by *t*-test (*P*<0.05) (*n*=12; n.s., non-significance). Error bar, s.d.

To investigate the effect of high pressure on a somatic cell, MEFs were harvested by trypsin treatment and hydrostatic pressures of 15, 30, 60, and 90 MPa were applied in the non-attached state. Although physiological range of hydrostatic pressure is 0.1–10 MPa, we chose this pressure range because cartilage may experience hydrostatic pressures around 20 MPa (Hodge et al., 1986; Afoke et al., 1987) and 40–80 MPa is used for pre-conditioning of embryos before vitrification (Siqueira Filho et al., 2011; Jiang et al., 2016). Hydrostatic pressure was applied for 10 min to detect the initial response of gene expression change. After application of the pressure, cells demonstrated high viability of 98.0±0.6%, 96.5±1.7%, 92.4±2.0% and 89.5±2.2% after 10 min application of 15 MPa, 30 MPa, 60 MPa and 90 MPa, respectively. When pressure applied cells were seeded and cultured, they attached to the culture dish and showed normal morphology within 6 h (Fig. 1A–E). These results indicate that hydrostatic pressure up to 60 MPa does not significantly impact the cell state, at least to the level that influences cell survival. On the other hand, the hydrostatic pressure of 90 MPa reduced the viability (*P*<0.05 compared to 15 MPa), suggesting that extremely high pressure may harm the cells. Close observation revealed multinucleated cells in 90 MPa applied cells (Fig. 1F, arrows). To quantify this observation, we counted the number of multinucleated cells and found that cells subjected to 60 and 90 MPa hydrostatic pressure had more multinucleated cells than control cells whereas no significant change was observed at 15 and 30 MPa (Fig. 1G), indicating that high hydrostatic pressure of >60 MPa might induce cell division failure.

**Fig. 2.**
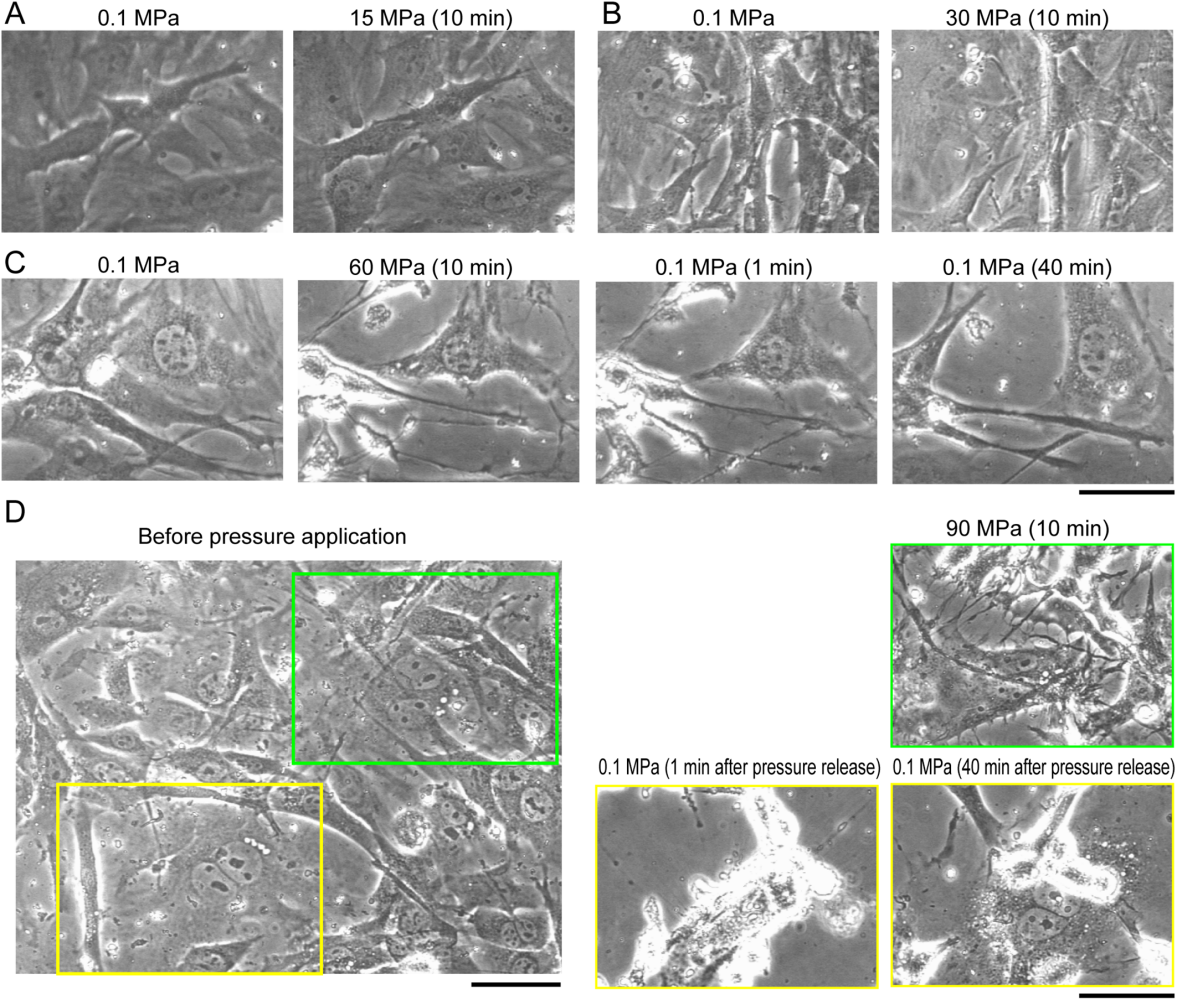
**Phase contrast images of MEF cells before and after the pressure treatment.** (A,B) Cells before (left) and after 10 min (right) application of hydrostatic pressure are shown. Cells were pressurized to 15 MPa (A), and 30 MPa (B). (C) Cells before application of pressure (left most), 10 min after 60 MPa applications of pressure (middle left), 1 min after release of pressure (middle right), and 40 min after release of pressure (right most). (D) Cells before application of pressure (left), 10 min after 90 MPa applications of pressure (right above, green), 1 min after release of pressure (middle, yellow), and 40 min after release of pressure (right below, yellow). Green and yellow rectangles indicate positions where images after application and release of pressure are shown. Scale bars: 50 μm. Representative images of three experiments are shown.

To further assess the effect of high pressure on the fibroblasts, we observed the morphological change during application of hydrostatic pressure on MEF cells, which were cultured on a glass surface, i.e. attached state. At low pressure levels (15 MPa and 30 MPa), morphological differences were negligible even when pressure was applied for 10 min; however, retraction of filopodia was observed (Fig. 2A,B). On the other hand, application of higher pressure (i.e. 60 MPa and 90 MPa) resulted in a drastic change in cell morphology (Fig. 2C,D). Retraction of filopodia and lamellipodia was observed immediately after the application of pressure, and the cells detached from the glass surface (Movie 1, 2). When cells that were pressurized to 60 MPa for 10 min were depressurized, they gradually regained their morphology (Movie 3). To our surprise, when cells that were pressurized to 90 MPa for 10 min were depressurized, cells suddenly shrank, resulting in protuberant cell shape (Fig. 2D, 1 min after pressure release; Movie 4). Cell edge became extremely bright due to shrinking by partial detachment of cells as phase contrast image increase its intensity as thickness of the cell increase. Although cells underwent drastic changes in morphology, cells started to spread when cultured at 0.1 MPa (Fig. 2D, 40 min after pressure release; Movie 5).

Changes in cell morphology upon application of hydrostatic pressure have been previously reported, which are caused by disruption of the cytoskeletal network (Bourns et al., 1988; Nishiyama, 2017). However, cells pressurized to 90 MPa underwent drastic changes in morphology when pressure was released. It is probable that even if the cytoskeletal network was disrupted under high pressure, cells maintained their shape by attachment to the substrate, which was detached by sudden drop in the pressure.

### Effect of hydrostatic pressure on gene expression

**Fig. 3.**
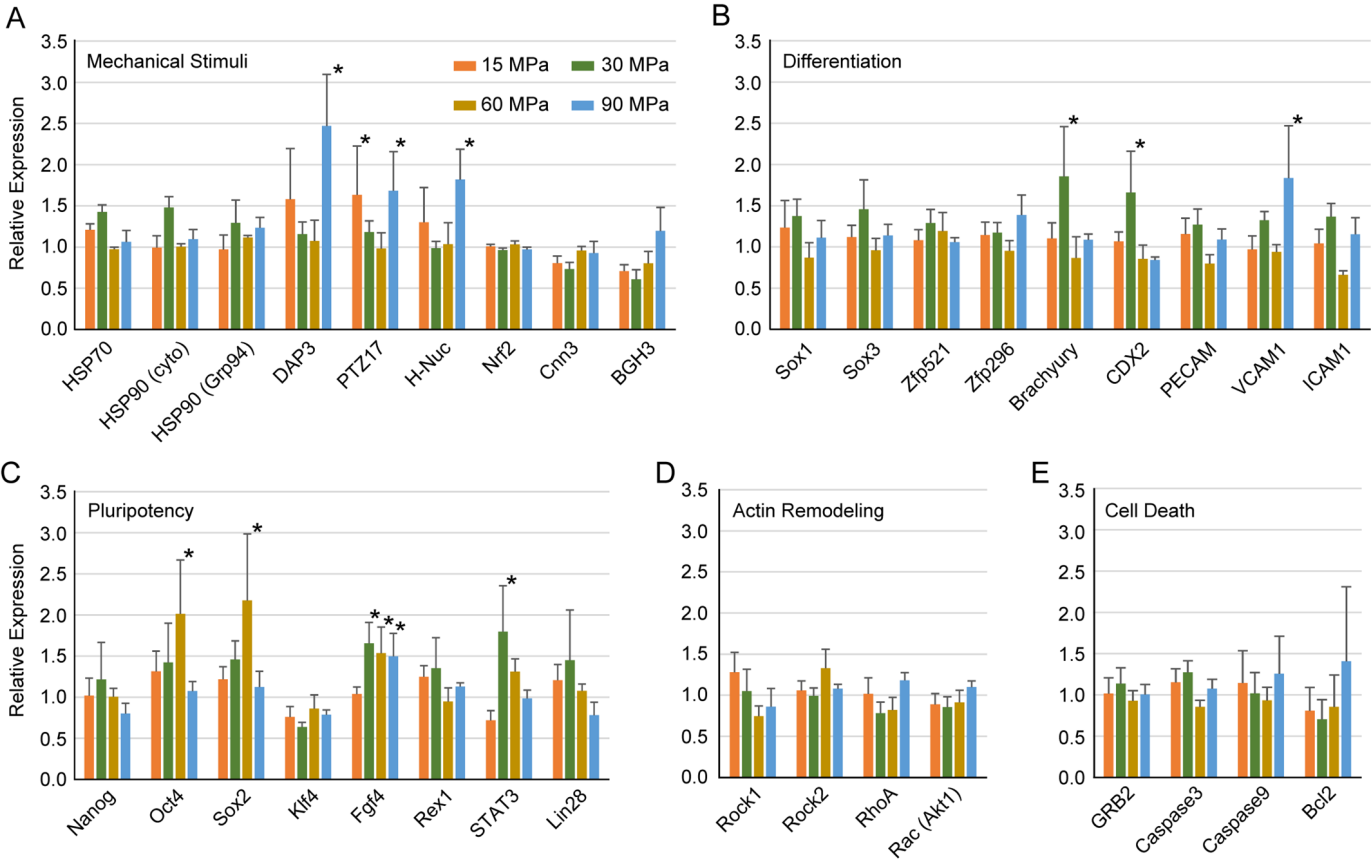
**Gene expression of MEF cells just after application of hydrostatic pressure.** Values are indicated in relative to cells without application of pressure. Genes related to mechanical stimuli (A), cell differentiation (B), genes related pluripotency (C), actin filament remodeling (D), and cell death (E) are investigated. Error bar show s.e. for five independent experiments. Asterisks show significance change by *t*-test (*P*<0.05) compared with cells without pressure treatment.

To further examine the effect of hydrostatic pressure on fibroblasts, we evaluated the change in gene expression by application of hydrostatic pressure. MEF cells were harvested by trypsinization and hydrostatic pressures of 15, 30, 60, and 90 MPa were applied for 10 min. mRNA was sampled after the pressure was released. Among genes that change their expression by mechanical stimuli, only *DAP3* was upregulated more than twofold at 90 MPa. *PTZ17* and *H-Nuc* were upregulated by 1.5 times at 90 MPa (Fig. 3A). It has been reported that *DAP3*, *PTZ17*, and *H-Nuc* were upregulated by hydrostatic pressure in chondrocytes (Sironen et al., 2000), indicating a similar pressure sensing mechanism between chondrocytes and fibroblasts. Because mechanical stimuli enhance differentiation (Kang et al., 2011; Zhao et al., 2015a; Lin et al., 2019), we examined genes that are related to differentiation, but none of the genes we tested were upregulated more than twofold. *Brachyury* and *CDX2* were upregulated more than 1.5 times at 30 MPa, and *VCAM1* was upregulated by over 1.5-fold at 90 MPa (Fig. 3B).

Next, we examined genes related to pluripotency (Fig. 3C) because the morphological change via actin disruption possibly relates to pluripotency (Higuchi et al., 2014). Surprisingly, *Oct4* and *Sox2* were upregulated more than twofold at 60 MPa. *Fgf4* was upregulated by more than 1.5 times between 30 and 90 MPa, and *STAT3* was upregulated more than 1.5 times at 30 MPa. However, other pluripotency-related genes such as *Nanog*, *Klf4*, *Rex1*, and *Lin28* did not change significantly. Genes related to actin filament remodeling and cell death did not change significantly (Fig. 3D,E).

The above results show that even cells were subjected to extremely high hydrostatic pressure and changes in cell morphology were observed, most of the genes we tested did not show significant changes in their expression. However, genes related to pluripotency were slightly upregulated by hydrostatic pressure.

### Effect of hydrostatic pressure on pluripotency related genes

**Fig. 4.**
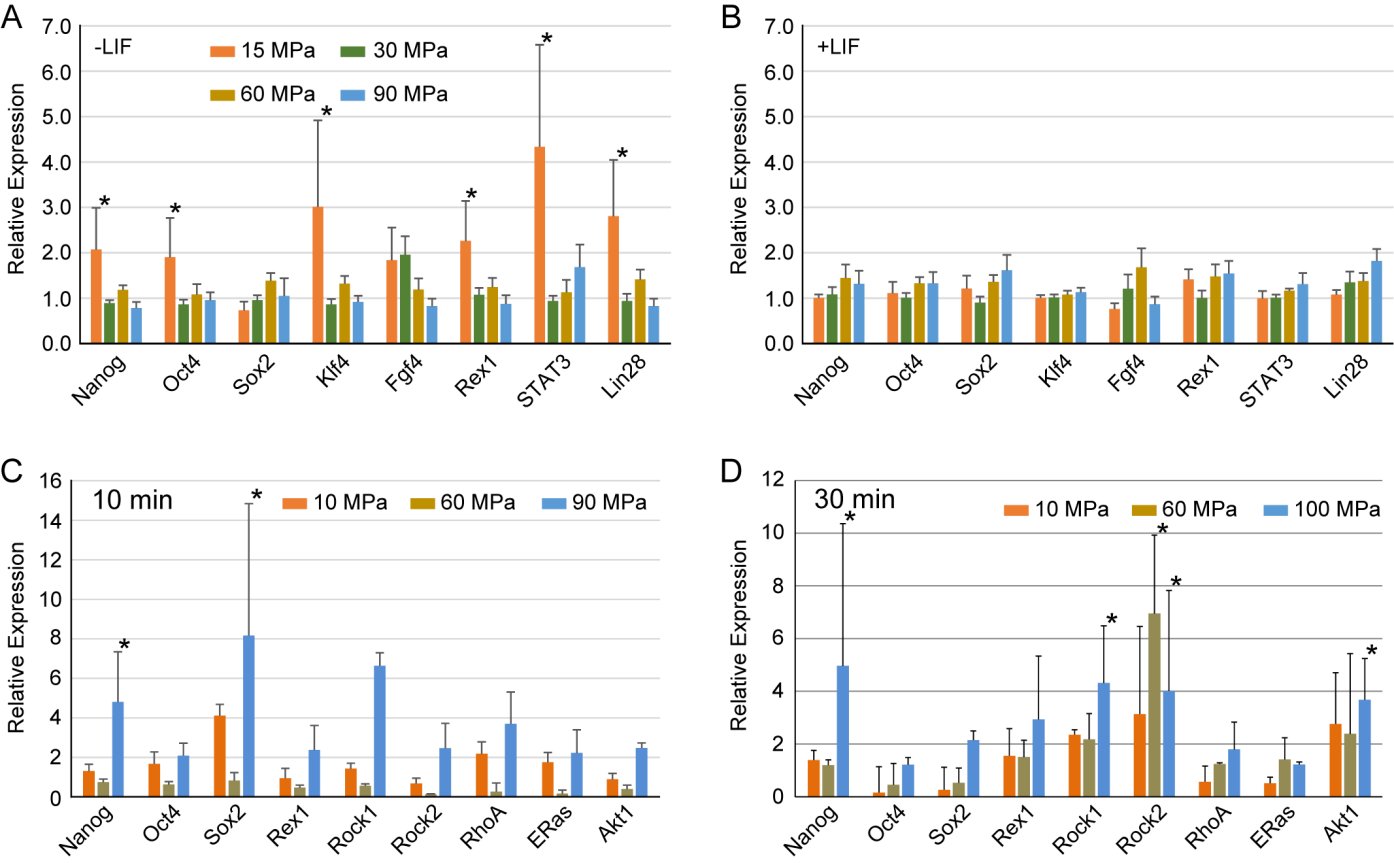
**Pluripotency gene expression of hydrostatic pressure applied to MEF (A,B) and mouse ES cells (C,D) after overnight culture.** For MEF, cells were cultured in the absence (A) or presence (B) of LIF. Hydrostatic pressure was applied for 10 min. For mouse ES cells, they were treated with hydrostatic pressure for 10 min (C) or 30 min (D) and cultured overnight in the presence of LIF. Error bars show s.e. for five independent experiments. Asterisks show significance change by *t*-test (*P*<0.05) compared with cells without pressure treatment.

Since pluripotency-related genes were slightly upregulated by application of hydrostatic pressure, we tested whether these genes were still upregulated after overnight culture. Of the pluripotency genes examined, *Nanog*, *Klf4*, *Rex1*, *STAT3*, and *Lin28* were upregulated more than twofold after 15 MPa application of hydrostatic pressure after overnight culture (Fig. 4A). However, although pluripotency genes were mostly upregulated at 60 MPa just after application of hydrostatic pressure, no significant changes were observed after overnight culture at this pressure. This result indicates that the changes observed just after application of pressure are only transient changes irrelevant to long-lasting changes in gene expression. Although some pluripotency genes were upregulated by 15 MPa and overnight culture, addition of LIF did not enhance this effect (Fig. 4B), indicating that cells were no longer in the pluripotency state.

### Hydrostatic pressure on ES cells

Although we found an increase in pluripotency genes after hydrostatic pressure application because the initial expression level of pluripotency genes in MEFs was small, the absolute change in these cells was also small. To test whether pluripotency genes could be upregulated in cells where these genes were already expressed, we applied pressure to mouse ES cells (Fig. 4C,D). A significant increase in *Sox2* expression was observed when ES cells were treated at 10 MPa for 10 min and *Nanog* expression increased more than twofold at 90 MPa. Increasing the duration to 30 min did not enhance the effect, where *Oct4* and *Sox2* expression decreased compared to the 10-min pressure application. However, expression of *Rock2* increased significantly after 30 min of pressure application compared with 10 min. PI3Ks (phosphatidylinositol 3 kinases) and their downstream mediator Akt1 are required for self-renewal of ES cells, and Pl3K signaling is activated by ES cell-specific RAS protein (ERas). It has already been suggested that an increase in Akt1 may inhibit GSK3 signaling, thus promoting pluripotency. In addition, ERas may have a role in the commitment of cell fate rather than maintaining pluripotency, as demonstrated by knockout and over-expression experiments (Takahashi et al., 2003; Hishida et al., 2015; Zhao et al., 2015b; Yu and Cui, 2016). The expression levels of Akt1 after 10 min of pressure application were almost unchanged. Increasing the duration to 30 min enhanced the expression levels of Akt1, which was expressed more than twofold. The expression levels of ERas did not significantly change with various hydrostatic pressure conditions at 10 min and 30 min duration. These results show that even in cells that express a substantial amount of pluripotency genes, hydrostatic pressure enhances their expression. In conclusion, we tested the effect of hydrostatic pressure application on mammalian cells and found that not only genes related to mechanical stimuli but also genes related to pluripotency were upregulated. These results indicate that hydrostatic pressure on mammalian cells may enhance cell reprogramming.

## MATERIALS AND METHODS

### Cell culture

The MEFs were isolated from the fetuses of 14 day pregnant BALB/c mice and cultured in Dulbecco's modified Eagle's medium (DMEM; Sigma-Aldrich, St. Louis, MO, USA) supplemented with 10% fetal bovine serum (FBS; Biowest, Miami, FL, USA) and 4 mM L-glutamine. Animal experiments were carried out according to the ethics guidelines of Kyoto University. Mouse ES cells (Riken Cell Bank, Ibaragi; E14Tg2a) were cultured in DMEM (Sigma-Aldrich, USA, D6046) containing 10% FBS (Gibco, USA, 16141-075), 1% penicillin-streptomycin (Sigma-Aldrich, P4333), 1% GlutaMAX-1 (Gibco, 35050-001), 1% non-essential amino acid (Gibco, USA, 11140-050), 1% nucleosides (Millipore, USA, ES-008-D), 1% sodium pyruvate (Sigma-Aldrich, S8636), 0.1% 2-mercaptoethanol (Sigma-Aldrich), and 0.1% leukemia inhibitory factor (LIF) (Nacalai, JP, NU0013-1), on 0.1% gelatin-coated 10 cm dishes (BD Biosciences, 353003) without feeder layers.

### High hydrostatic pressure treatment on cells for quantitative PCR measurements

We designed a pressure device that could apply pressure up to 200 MPa to cells in solution. The device was composed of a high-pressure chamber (Syn Corporation Ltd, Kyoto, Japan) with a piezometer (Uinics Co., Ltd, Osaka, Japan) and an electrical compressor (Max Co., Ltd, Tokyo, Japan). The high-pressure chamber could maintain the applied pressure for 8 h. All parts of the device were assembled by Syn Corporation (Kyoto, Japan). Cells were trypsinized and dissociated into single cells by pipetting. 1×10^5^ cells were placed in a 2 ml tube where the lids were cut off, and the culture medium was filled to the top of the tube to exclude air. Then, the top of the tubes was completely sealed with Parafilm (Bemis Flexible Packaging, Oshkosh, WI, USA). The tubes were placed in a high-pressure chamber, and then hydrostatic pressure of 15, 30, 60 and 90 MPa was applied. After release of pressure, the tubes were removed from the chamber, and cells were collected for RNA isolation.

### Quantitative PCR

Total RNA was isolated using the RNeasy Mini kit (Qiagen, Germany) according to the manufacturer's instructions. The total RNA was then reverse transcribed to cDNA using an Omniscript RT kit (Qiagen) according to the manufacturer's instructions. Real-time quantitative PCR (RT-qPCR) was performed on a CFX 96 Real Time system (Bio-Rad, Hercules, CA, USA) using Thunderbird SYBR PCR Master Mix (TOYOBO, Japan). The cycling conditions were 98°C for 3 min, followed by 30 s at 95°C, 30 s at 55°C, and 33 cycles with gene-specific primers. The raw values were normalized to the expression levels of several housekeeping genes as endogenous controls. The gene-specific primers used for this analysis are presented in Table S1.

### High-pressure microscopy

The details of the high-pressure microscope have been previously described (Nishiyama and Sowa, 2012). The high-pressure microscope was optimized for both the best image formation and the stability to hydrostatic pressure up to 150 MPa. A miniature glass bottom dish was inserted into the chamber. The miniature glass bottom dish was constructed with a round cover glass (thickness=0.17 mm, diameter=6 mm), plain washer (stainless steel; diameter=6 mm), and strip of double-faced adhesive tape (1510, 3 M). The surface of the cover glass was coated with 0.1% gelatin for 30 min and MEFs were seeded one day before the observation.

Hydrostatic pressure of 15, 30, 60 and 90 MPa was applied using the hand pump and then decreased by opening the valve. The inner temperature of the chamber was 36±1°C, which was controlled by running temperature-regulated water from a thermostat bath. Microscopic observations were carried out using a long-working-distance objective lens (CFI S Plan Fluor ELWD ADM 20×, Nikon, Japan), and the phase-contrast images were recorded with a charge-coupled device camera (WAT-120N+, Watec, Tsuruoka, Japan). All microscopic images were stored in a computer and analyzed offline. The brightness was adjusted linearly for easy viewing.

### Statistical analysis

Statistical analysis of data was performed using unpaired Student's two-sided *t*-test using Excel (Microsoft, Redmond, WA, USA).

## Supplementary Material

Supplementary information
