# Supplementary material for: Pressure-induced changes on the morphology and gene expression in mammalian cells
Source: Biol Open. 2021 Jul 14;10(7):bio058544. doi: 10.1242/bio.058544 (PMC8325925; doi:10.1242/bio.058544)
Supplement: Supplementary information [file biolopen-10-058544-s1.pdf]

Table S1: Primers used for quantitative PCR

Genes	Forward	Reverse
HSP70	CTGGCTCAGGACCCACCATC	CTTGACAGTAATCGGTGCCCA
HSP90 (cyto)	CCCCAGACCCATGCTAACAG	TGCGTGATGTGTCGTCATCT
HSP90 (Grp94)	AGACATGTTGCGGCGGATTA	TCTCCATACGCCTTGGTGTC
DAP3	TGTCCTACAGCTAACACCAGT	GCCAGCAAACAGAACACCAG
PTZ17	AGAGTGTGAGAGGCTGTCCT	CTAAGTCAGGCAGGTGAGGC
H-Nuc	GAACCTGTCCAGGCTGCTAT	AGCGGTAGTAACAGGTTGCC
Nrf2	CCGCCCAGAACTGTAGGAAA	AGATACAAGGTGCTGAGCCG
Cnn3	GGCTGAAGGACGGCATCATA	TGGGGCTTCATACCGTAAGC
BGH3	CTGTTGCCGAAACCGACATC	ATACGCTGACGCCTGTTTGA
Sox1	CCTCGGATCTCTGGTCAAGT	GCAGGTACATGCTGATCATCTC
Sox3	CACAACTCCGAGATCAGCAA	TCCGGGTACTCCTTCATGTG
Zfp521	GAGCGAAGAGGAGTTTTTGG	AGTTCCAAGGTGGAGGTCAC
Zfp296	GTGACCAGTGTCCCTATGCTT	CCATTTCCACTTGCTTTAGTGA
Brachyury	CAAGACTCCTGGAAGGTGGAGAG	TTCGCGTTCGGTGGGGT
CDX2	CTTTGTCAGTCCTCCGCAGT	CGTAGCCATTCCAGTCCTCG
PECAM	TCAGCTGCCAGTCCGAAAATGG	TCTGGAAGTCACTCTTTGCTTT
VCAM1	GTTGTAACTTAATGCTTCTTAG	GAACCCTGACTTCTTTTAAA
ICAM1	AGGAGGTGAATGTATAAGTT	GTCGACCCTTATGAGAAAAGC
Nanog	AGGGTCTGCTACTGAGATGCTCTG	CAACCACTGGTTTTTCTGCCACCG
Oct4	TCTTTCCACCAGGCCCCCGGCTC	TGCGGGCGGACATGGGGAGATCC
Sox2	TAGAGCTAGACTCCGGGCGATGA	TTGCCTTAAACAAGACCAGGAAA
Klf4	AGTTCTCCAACTGCTGCATACT	AACACATTTGTTCTGCTTAA
Fgf4	AAGACGGTCTCATAGCCCAGGCT	AGCAGTAGGCCAGCCTTGGCT
Rex1	ATTCCATGGTATAGTTCCAACAGG	TGTTGACTACTGCCAAAGTTGGCC
STAT3	CTTGTCTACCTCTACCCCGACAT	GATCCATGTCAAACGTGAGCG
Lin28	CCAATGTAATCTGTCACCCTTAG	GCTTTGGTTTCTAGCAGTAAACA
Rock1	TTCATGTCCGACCTGTAACC	TTGACAGCGTTCGAGGAGAG
Rock2	TTCACGTCCGACCTGTTACC	GTGGCACCTACGGCACTCTA
RhoA	TTCGGAATGACGAGCACACG	GTCTAGCTTGCAGAGCAGCT
Rac (Akt1)	GTCCAGGGCCAAAGTCCAGCA	CCTAAATGTCTCTGAACAGCATG
GRB2	CAGTGGAATTAAAAAGGGTGGCA	TTGGCGATGGCTTCCATTCT
Caspase3	GCGTGACCGGCGGTAGT	GTCCAGATAGATCCCAGAGTCCA
Caspase9	GGATCCCTGGAGGCGCTATG	CCTGCCTGCTGAATATCCTCG
Bcl2	TCTTTGAGTTCGGTGGGGTC	AGTTCCACAAAGGCATCCCAG

Biology Open • Supplementary information

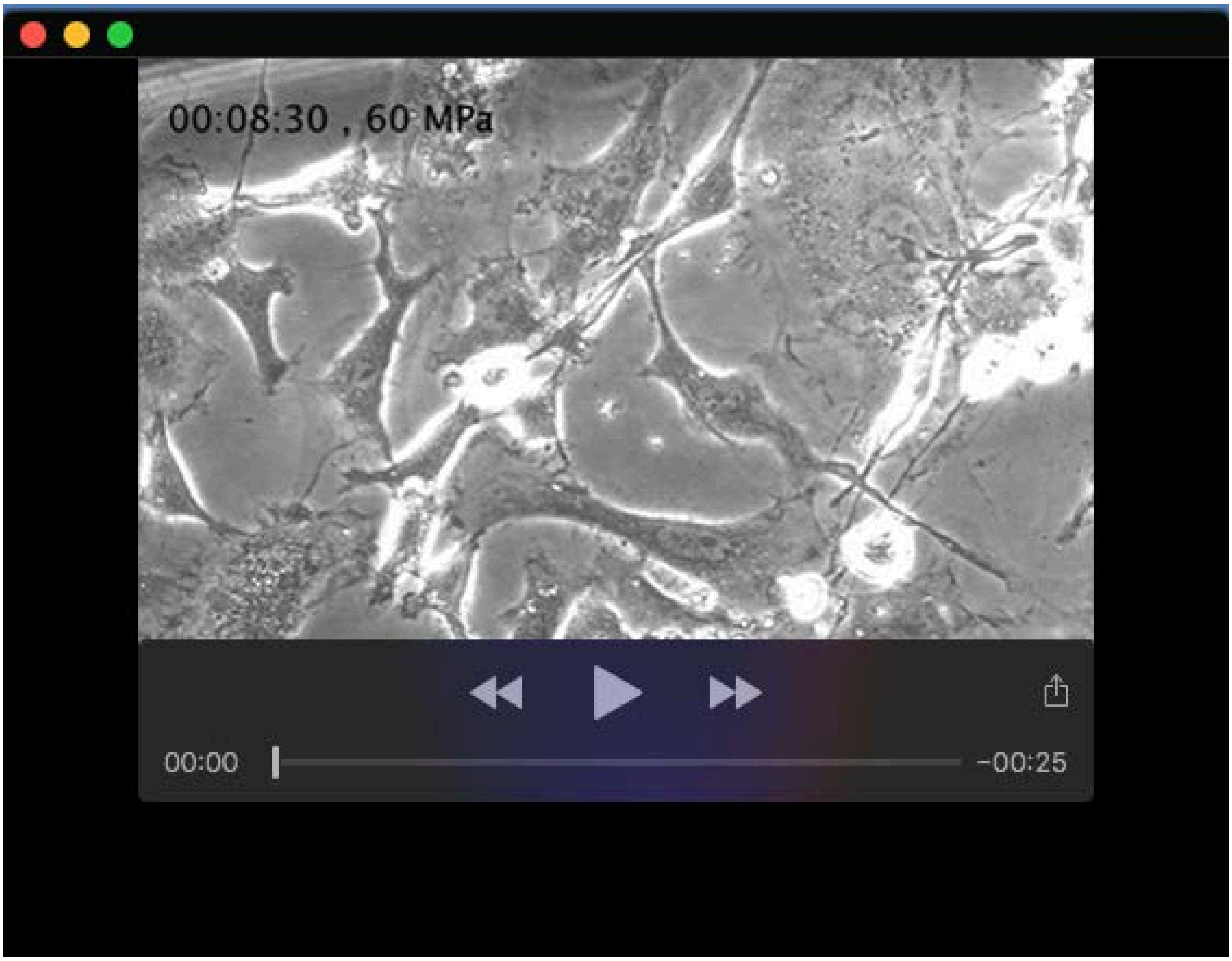

Movie 1. Phase contrast movie of MEF cells just after the application of 60 MPa hydrostatic pressure. MEF cells were attached to coverslip day before observation and pressure was applied just before the beginning of the movie. Total length of this movie is 10 min. Scale bar, 50 µm.

Biology Open • Supplementary information

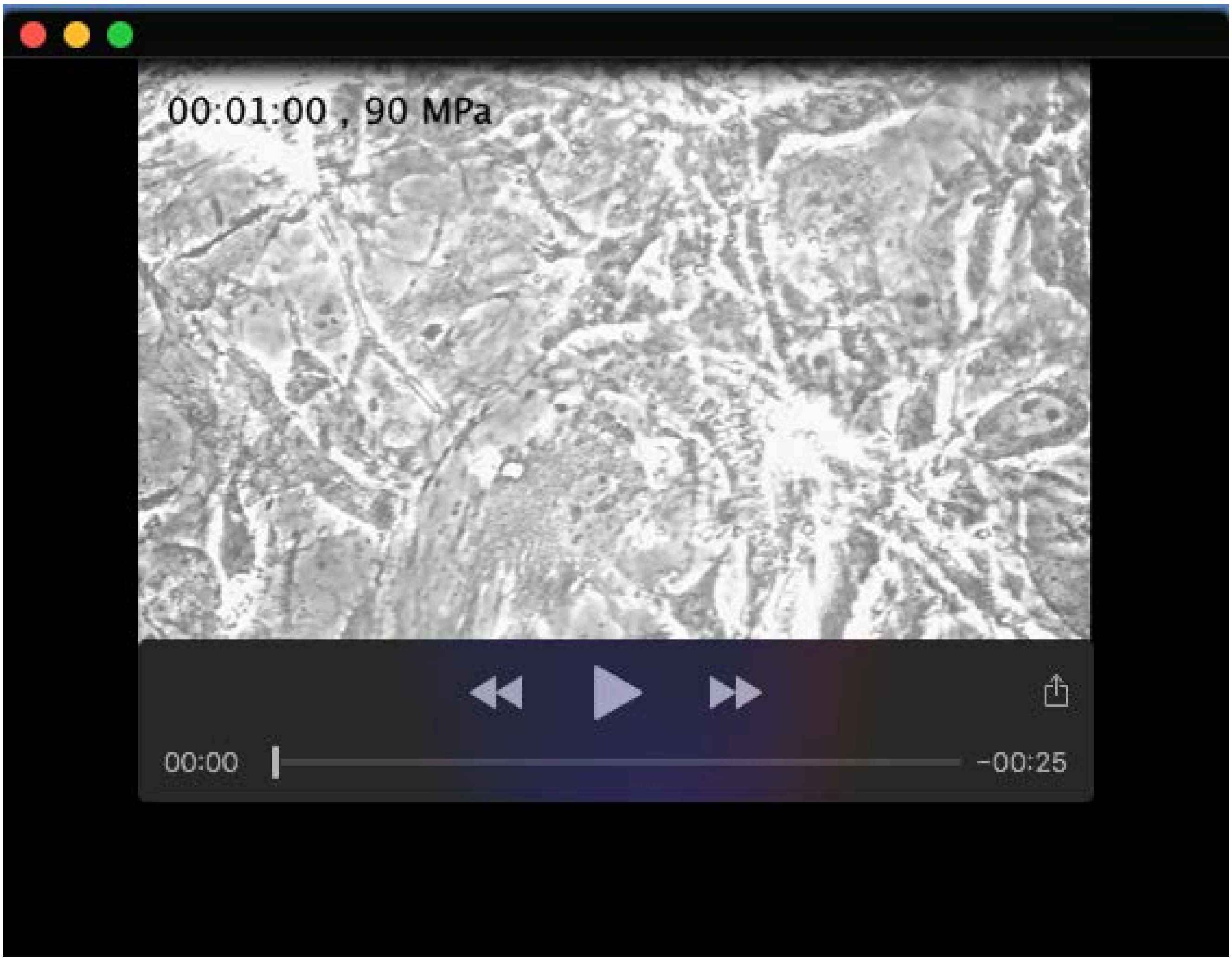

Movie 2. Phase contrast movie of MEF cells just after the application of 90 MPa hydrostatic pressure. MEF cells were attached to coverslip day before observation and pressure was applied just before the beginning of the movie. Total length of this movie is 10 min. Scale bar, 50 µm.

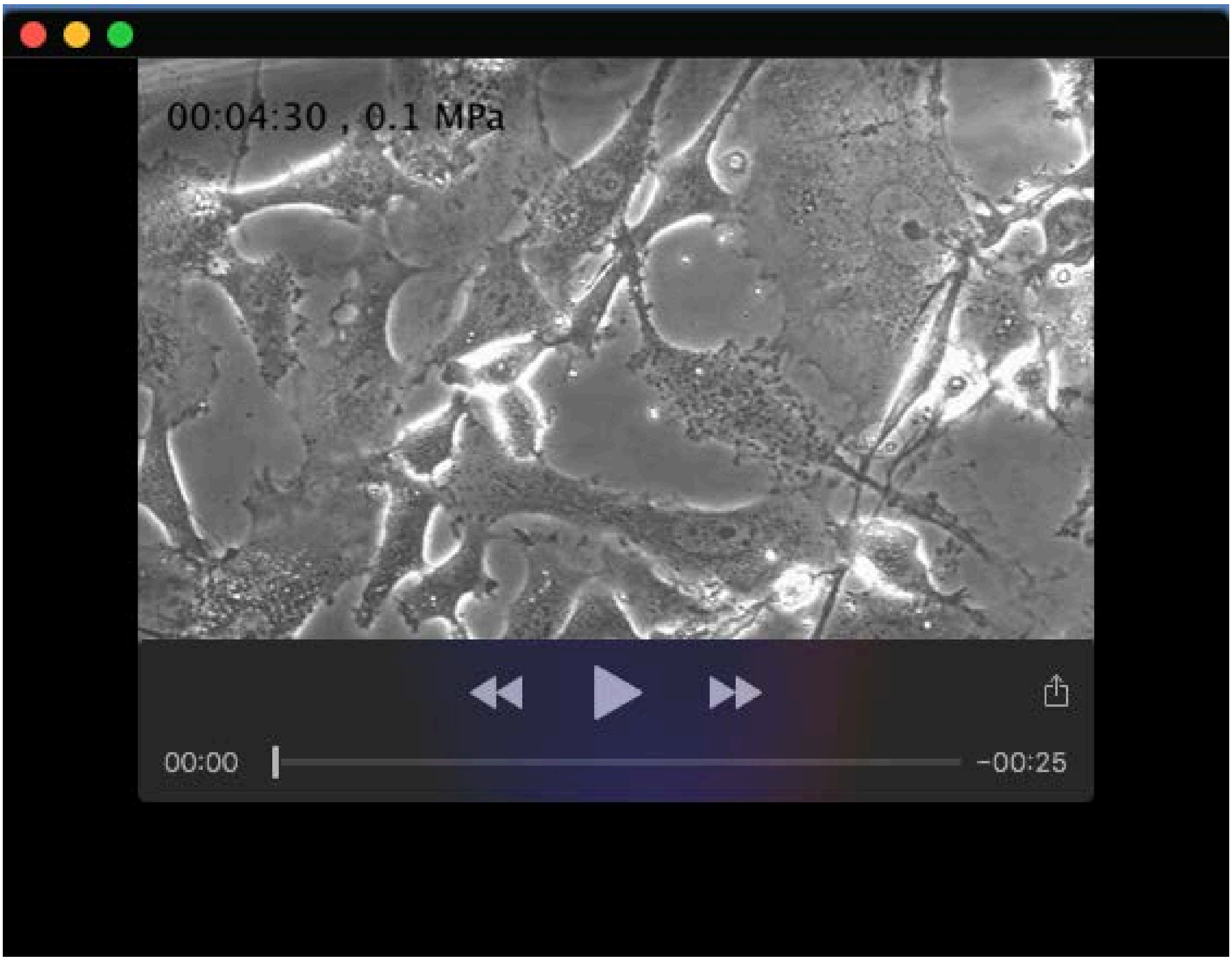

Movie 3. Phase contrast movie of MEF cells just after the release of hydrostatic pressure. Hydrostatic pressure of 60 MPa was applied to MEF cells for 10 min, and then the pressure was released to 0.1 MPa just before the beginning of the movie. Total length of this movie is 40 min. Scale bar, 50 µm.

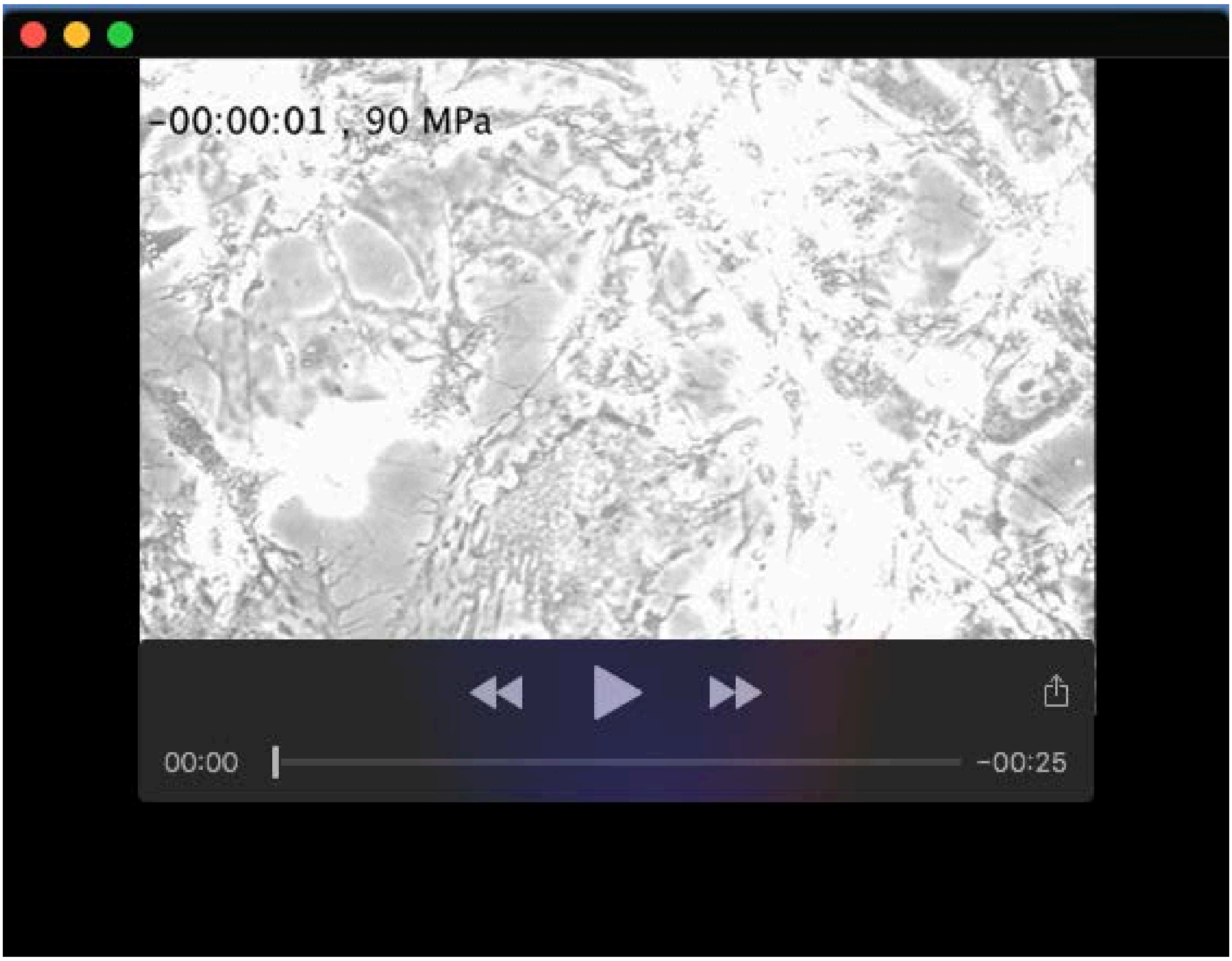

Movie 4. Phase contrast movie of MEF cells just before and after the release of hydrostatic pressure. Hydrostatic pressure of 90 MPa was applied for 10 min, and then the pressure was released to 0.1 MPa at time 0 as indicated in the movie. Scale bar, 50 µm.

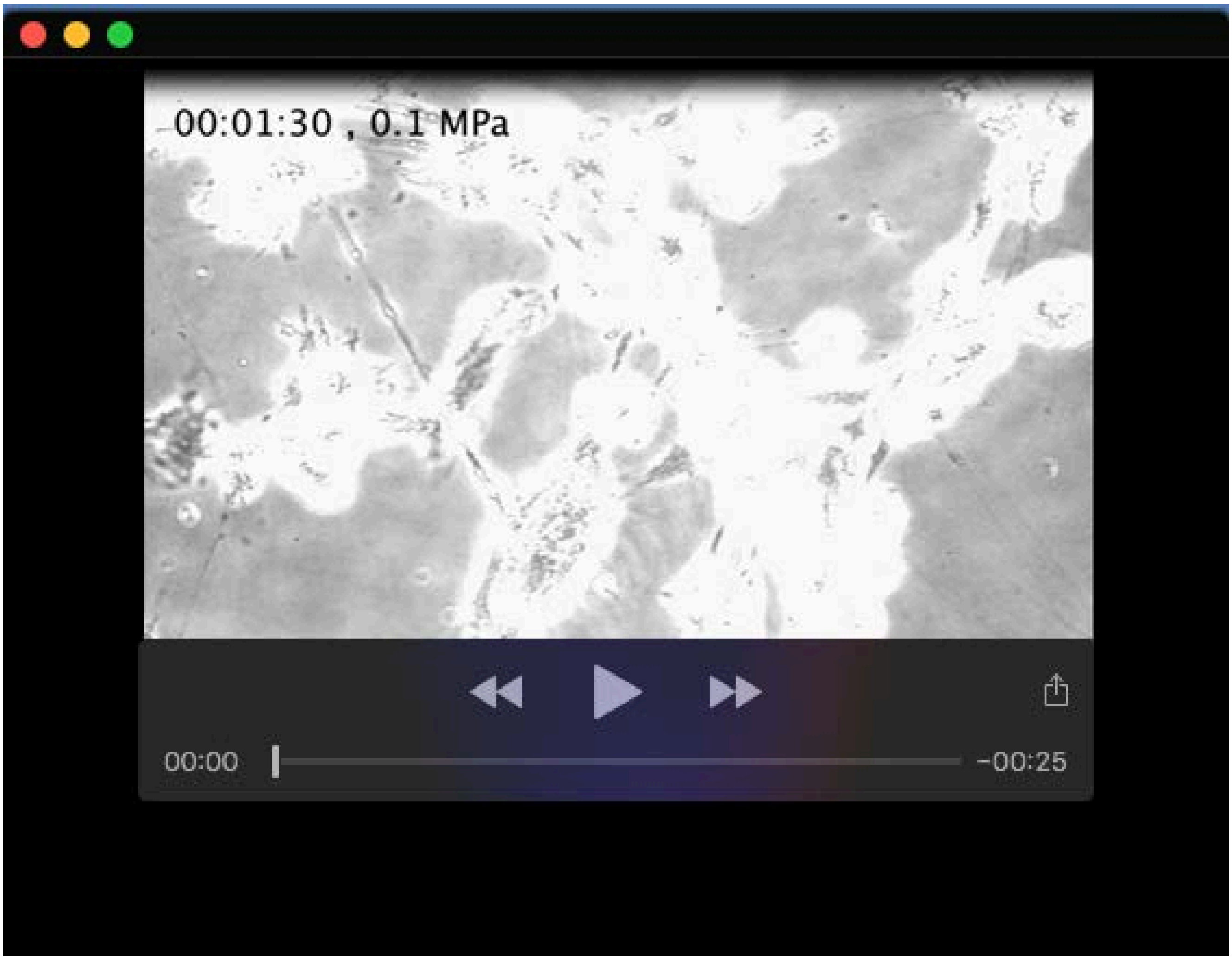

Movie 5. Phase contrast movie of MEF cells just after the release of hydrostatic pressure. Hydrostatic pressure of 90 MPa was applied to MEF cells for 10 min, and then the pressure was released to 0.1 MPa just before the beginning of the movie. Total length of this movie is 40 min. Scale bar, 50 μm.
